# Factors Associated With Public Trust in Pharmaceutical Manufacturers

**DOI:** 10.1001/jamanetworkopen.2023.3002

**Published:** 2023-03-14

**Authors:** Yashaswini Singh, Matthew D. Eisenberg, Neeraj Sood

**Affiliations:** 1 Department of Health Policy and Management, Johns Hopkins Bloomberg School of Public Health, Johns Hopkins University, Baltimore, Maryland; 2 Sol Price School of Public Policy, University of Southern California, Los Angeles

## Abstract

This cross-sectional study examines how key demographic and predisposing factors are associated with consumer trust in pharmaceutical manufacturers.

## Introduction

Public distrust in the pharmaceutical industry has increased, in part due to perceptions of pharmaceutical manufacturers as profit seeking and in part due to the actions of pharmaceutical manufacturers, including off-label marketing, overcharging government programs, and concealing data.^1^ Notwithstanding the underlying mechanism, an unfavorable public perception of pharmaceutical manufacturers is concerning if it translates into poor medication adherence,^2^ lack of participation in clinical trials,^3^ and rejection of effective health interventions, including vaccine campaigns.^4^ In this cross-sectional study, we examined how key demographic and predisposing factors are associated with consumer trust in pharmaceutical manufacturers.

## Methods

A nationally representative sample of individuals at high risk of cardiovascular disease (US residents aged 40-64 years, who currently smoke, with high cholesterol, or with a body mass index >25 [calculated as weight in kilograms divided by height in meters squared]) were recruited from the Ipsos Public Affairs LLC KnowledgePanel. Participants completed a survey fielded to 4933 respondents, of whom 3026 respondents started and completed the survey (response rate of 61%). Further details on the survey design are described in an earlier study using the same sample.^5^ Our cross-sectional study followed the Strengthening the Reporting of Observational Studies in Epidemiology (STROBE) reporting guideline and received institutional review board approval from the University of Southern California. All participants provided electronic informed consent.

The dependent variable measured individuals’ trust in pharmaceutical manufacturers on a 5-point Likert scale (ranging from 1 [always distrust] to 5 [always trust]). Independent variables included demographic characteristics (such as gender, race and ethnicity, age, and geographic location) and other predisposing factors, including political affiliation, household income, educational level, self-reported health, and source of health information (including whether they have a regular source of care and whether they rely on digital media for health information).

Characteristics of individuals with high and low levels of trust were compared using χ^2^ tests. All *P* values were from 2-sided tests and results were deemed statistically significant at *P* < .05. The association between demographic characteristics and trust was estimated using adjusted odds ratios (ORs) from an ordered logit regression model.

## Results

This cross-sectional study used a nationally representative survey of 2867 individuals at risk of cardiovascular disease (mean [SD] age, 54 [7] years; 1324 women [46%] and 2119 White individuals [74%]). A total of 1145 individuals (40%) considered pharmaceutical manufacturers to be sometimes (1036 [36%]) or always (109 [4%]) trustworthy (Table 1).

**Table 1.** Perceptions of Pharmaceutical Manufacturers by Individual Characteristics

Characteristic	No. (%)			*P* value^b^
	Total (N = 2867)	Low trust (n = 1722)^a^	High trust (n = 1145)^a^	
Female	1324 (46)	790 (46)	534 (47)	.69
White	2118 (74)	1276 (74)	842 (74)	.74
Age, mean (SD), y	54 (7)	54 (7)	54 (7)	.06
Political affiliation				
Democrat	779 (27)	426 (25)	353 (31)	<.001
Independent or other	1121 (39)	728 (42)	393 (34)	
Republican	956 (33)	561 (33)	395 (35)	
Household income, $				
10 000-24 999	289 (10)	183 (11)	106 (9)	.02
25 000-49 999	427 (15)	274 (16)	153 (13)	
50 000-74 999	439 (15)	269 (16)	170 (15)	
75 000-99 999	422 (15)	261 (15)	161 (14)	
>100 000	1290 (45)	735 (43)	555 (49)	
Educational level				
No high school diploma or GED	145 (5)	89 (5)	56 (5)	.09
High school graduate	770 (27)	462 (27)	308 (27)	
Some college or Associate’s degree	940 (33)	587 (34)	353 (31)	
Bachelor’s degree	593 (21)	356 (21)	237 (21)	
Master’s degree or higher	419 (15)	228 (13)	191 (17)	
Used the internet, television, or social media as a source of health information	1447 (51)	849 (49)	598 (52)	.13
Had a usual source of health care in the past year	2226 (78)	1306 (76)	920 (80)	.005
Self-reported health				
Excellent	175 (6)	97 (6)	78 (7)	.008
Very good	950 (33)	545 (32)	405 (35)	
Good	1242 (43)	749 (44)	493 (43)	
Fair	417 (15)	270 (16)	147 (13)	
Poor	81 (3)	59 (3)	22 (2)	
Rural	579 (20)	348 (20)	231 (20)	.98
Region				
Northeast	538 (19)	295 (17)	243 (21)	.03
Midwest	638 (22)	389 (23)	249 (22)	
South	1059 (37)	638 (37)	421 (37)	
West	632 (22)	400 (23)	232 (20)	

Abbreviation: GED, General Educational Development certification.

^a^ Trust in pharmaceutical manufacturers was measured using a survey question that asks about individuals’ trust in pharmaceutical manufacturers on a 5-point Likert scale (ranging from 1 [always distrust] to 5 [always trust]). Of 2874 individuals, 167 (6%) reported always distrusting pharmaceutical manufacturers, 766 (27%) reported sometimes distrusting, 789 (27%) reported neither trusting nor distrusting, 1036 (36%) reported sometimes trusting, and 109 (4%) reported always trusting pharmaceutical manufacturers. “High” trust represents individuals who report that they sometimes or always trust pharmaceutical manufacturers.

^b^ Results from χ^2^ tests.

Excellent health (OR, 1.70 [95% CI, 1.05-2.75]; *P* = .03) and having a regular source of care (OR, 1.19 [95% CI, 1.01-1.40]; *P* = .03) were associated with higher trust in pharmaceutical manufacturers (Table 2). Individuals with Democratic (OR, 1.35 [95% CI, 1.15-1.61]; *P* < .001) or Republican party affiliation (OR, 1.27 [95% CI, 1.09-1.49]; *P* = .003) had higher trust relative to those with Independent affiliation. Relative to the west, individuals in the northeast had higher trust (OR, 1.43 [95% C, 1.16-1.77]; *P* = .001). There were no differences across gender, race and ethnicity, age, income, or educational level.

**Table 2.** Adjusted Associations Between Individual Characteristics and Trust in Pharmaceutical Manufacturers

Characteristic	Adjusted odds ratio (95% CI)^a^	*P* value
Female	1.01 (0.88-1.16)	.87
Race and ethnicity		
White	0.87 (0.74-1.02)	.09
Non-White^b^	1 [Reference]	
Age	1.01 (0.99-1.02)	.12
Educational level		
Bachelor’s degree or higher	0.96 (0.82-1.13)	.66
Some college, high school, or GED	1 [Reference]	
Political affiliation		
Democrat	1.35 (1.15-1.61)	<.001
Republican	1.27 (1.09-1.49)	.003
Independent or other	1 [Reference]	NA
Household income, $		
<24 999	1 [Reference]	NA
25 000-49 999	0.78 (0.59-1.02)	.09
50 000-74 999	0.99 (0.79-1.24)	.96
75 000-99 999	1.01 (0.80-1.26)	.92
>100 000	1.20 (0.99-1.45)	.06
Used the internet, television, or social media for health information	0.92 (0.81-1.06)	.29
Had a usual source of health care in the past year	1.19 (1.01-1.40)	.03
Self-reported health		
Excellent	1.70 (1.05-2.75)	.03
Very good	1.48 (0.98-2.24)	.06
Good	1.38 (0.92-2.06)	.11
Fair	1.21 (0.79-1.86)	.35
Poor	1 [Reference]	NA
Region		
Northeast	1.43 (1.16-1.77)	.001
Midwest	1.17 (0.96-1.44)	.11
South	1.15 (0.96-1.37)	.18
West	1 [Reference]	NA

Abbreviations: GED, General Educational Development certification; NA, not applicable.

^a^ Estimates represent adjusted odds ratios from an ordered logit regression with pharmaceutical trust as the dependent variable. Pharmaceutical trust is measured on a 5-point Likert scale (ranging from 1 [always distrust] to 5 [always trust]).

^b^ Included individuals who self-reported race and ethnicity as Hispanic, Asian, Black, 2 or more races, or other.

## Discussion

Approximately 60% of individuals at high risk for cardiovascular disease did not trust pharmaceutical manufacturers. Lack of trust was higher among those in poor health or without a usual source of care, raising concerns that vulnerable populations have experiences where trust has been broken. This also raises concerns about poor medication adherence and lack of treatment-seeking behavior in vulnerable populations. Those with Independent political affiliation had lower trust than those with Republican or Democratic affiliation, suggesting that mainstream political discourse might be associated with pharmaceutical trust. However, those with Independent affiliation might represent different ideological backgrounds. There were significant regional differences, with those in the northeast, where several pharmaceutical firms have a major presence, having higher trust. Limitations to this study include limited generalizability, cross-sectional design, and self-reported data in a survey-based design with potential for nonresponse bias.

## Notes

Supplement.
Data Sharing Statement
