# Supplementary material for: Factors Associated With Public Trust in Pharmaceutical Manufacturers
Source: JAMA Netw Open. 2023 Mar 14;6(3):e233002. doi: 10.1001/jamanetworkopen.2023.3002 (PMC10015300; doi:10.1001/jamanetworkopen.2023.3002)
Supplement: Supplement. — Data Sharing Statement [file jamanetwopen-e233002-s001.pdf]

Data Sharing Statement

Singh. Factors Associated With Public Trust in Pharmaceutical Manufacturers. *JAMA Netw Open*. Published March 14, 2023. doi:10.1001/jamanetworkopen.2023.3002

Data
Data available: No
